# Development of a Short and ICD-11 Compatible Measure for *DSM-5* Maladaptive Personality Traits Using Ant Colony Optimization Algorithms

**DOI:** 10.1177/1073191120971848

**Published:** 2020-12-28

**Authors:** André Kerber, Martin Schultze, Steffen Müller, Rosa Maria Rühling, Aidan G. C. Wright, Carsten Spitzer, Robert F. Krueger, Christine Knaevelsrud, Johannes Zimmermann

**Affiliations:** 1 Freie Universität Berlin, Berlin, Germany; 2 Goethe University Frankfurt, Frankfurt am Main, Germany; 3 University of Kassel, Kassel, Germany; 4 University of Pittsburgh, Pittsburgh, PA, USA; 5 University Medical Center Rostock, Rostock, Germany; 6 University of Minnesota, Minneapolis, MN, USA

**Keywords:** PID-5, *DSM-5*, ICD-11, screening tool, maladaptive personality traits, ant colony optimization, personality disorder

## Abstract

While *Diagnostic and Statistical Manual of Mental Disorders–Fifth edition* (*DSM-5*) Section III and ICD-11 (International Classification of Diseases 11th–Revision) both allow for dimensional assessment of personality pathology, the models differ in the definition of maladaptive traits. In this study, we pursued the goal of developing a short and reliable assessment for maladaptive traits, which is compatible with both models, using the item pool of the Personality Inventory for *DSM-5* (PID-5). To this aim, we applied ant colony optimization algorithms in English- and German-speaking samples comprising a total N of 2,927. This procedure yielded a 34-item measure with a hierarchical latent structure including six maladaptive trait domains and 17 trait facets, the “Personality Inventory for *DSM-5*, Brief Form Plus” (PID5BF+). While latent structure, reliability, and criterion validity were ascertained in the original and in two separate validation samples (*n* = 849, *n* = 493) and the measure was able to discriminate personality disorders from other diagnoses in a clinical subsample, results suggest further modifications for capturing ICD-11 Anankastia.

The classification and diagnosis of personality disorders (PD) is shifting away from categorical models toward a dimensional approach (Krueger & Markon, 2014; Mulder & Tyrer, 2018; Tyrer et al., 2018). In the *Diagnostic and Statistical Manual of Mental Disorders–Fifth edition* (*DSM-5*) Section III (American Psychiatric Association, 2013a), a dimensional Alternative Model for Personality Disorders (AMPD) has been added as an optional, “emerging model,” whereas in the ICD-11 (International Classification of Diseases 11th–Revision; World Health Organization, 2018) PD categories will be completely replaced by a dimensional model (Tyrer et al., 2018). This shift was motivated by notable limitations of categorical models including high comorbidity and low specificity of PD diagnoses, overreliance on “PD not otherwise specified,” and a generally poor match to the empirical covariation of PD criteria (Hengartner et al., 2018; Widiger & Trull, 2007). The emerging dimensional models aim to address these issues by incorporating individual differences in PD severity and style (Zimmermann et al., 2019).

To represent stylistic differences in the expression of PD, the *DSM-5* AMPD and the ICD-11 model include a set of maladaptive trait domains, although their definitions vary somewhat between the two diagnostic systems (Mulder & Tyrer, 2018). The *DSM-5* AMPD defines the five maladaptive trait domains Negative Affectivity, Detachment, Antagonism, Disinhibition, and Psychoticism. These in turn are composed of 25 facet traits, such as emotional lability or anxiousness for the Negative Affectivity domain or manipulativeness or grandiosity for the Antagonism domain. The ICD-11 model similarly includes five maladaptive trait domains, Negative Affectivity, Detachment, Dissociality, Disinhibition, and Anankastia, but does not define facet traits to facilitate the application of the model in clinical practice. To provide a self-report measure for the *DSM-5* AMPD trait model, the American Psychiatric Association published the Personality Inventory for *DSM-5* (PID-5; Krueger et al., 2012), which captures all 25 trait facets with 220 items. For the assessment of ICD-11 trait domains, the recently developed Personality Inventory for ICD-11 (PICD; Oltmanns & Widiger, 2018) and Five-Factor Personality Inventory for ICD-11 (FFiCD; Oltmanns & Widiger, 2020) are available.

A psychometric review of 39 studies using the PID-5 demonstrated high internal consistency for domain scores and acceptable consistency for trait facet scores across studies (Al-Dajani et al., 2016). A recent meta-analysis across 14 independent samples with *N* = 14,743 (Watters & Bagby, 2018) as well as a quantitative review including 23 studies based on 25 samples with *N* = 24,240 (Somma et al., 2019) confirmed the latent structure of the *DSM-5* AMPD trait domains and facets. Maladaptive personality traits according to *DSM-5* AMPD have been found to largely recover the PD categories and symptoms specified in the ICD-10 or *DSM-IV*, which could be ascertained in a meta-analysis with weaker coverage concerning obsessive compulsive PD (Watters, Bagby, et al., 2019). Furthermore, there is considerable evidence that the *DSM-5* AMPD traits can be conceived of as maladaptive variants of general personality traits, probably with the exception of Psychoticism, which is often rather unrelated to Openness (e.g., Gore & Widiger, 2013; Suzuki et al., 2015; Z. E. Wright et al., 2017; Zimmermann, Altenstein, et al., 2014). There is also a large body of research associating maladaptive traits according to the *DSM-5* AMPD with a range of transdiagnostic variables such as interpersonal problems, childhood maltreatment, maladaptive schemas, pathological beliefs, attachment anxiety and avoidance, emotion dysregulation and neuronal connectivity, suggesting their significant role in general psychopathology (for a comprehensive overview, see Zimmermann et al., 2019).

Studies using trait measures that were explicitly designed for the ICD-11 proposal (Oltmanns & Widiger, 2018, 2020) are still scarce but first findings suggest a strong correspondence between four maladaptive trait domains. In particular, the *DSM-5* trait domains Negative Affectivity, Detachment, Antagonism, and Disinhibition largely correspond to the ICD-11 trait domains Negative Affectivity, Detachment, Dissociality, and Disinhibition (McCabe & Widiger, 2020). In anticipation of these findings, Bach et al. (2017) constructed a “cross-walk” between *DSM-5* trait facets and ICD-11 trait domains using exploratory factor analysis of PID-5 facet scores, suggesting that the missing ICD-11 trait domain Anankastia could be assessed by the *DSM-5* trait facets “rigid perfectionism” and “perseveration.” Based on their findings, they developed an algorithm for the operationalization of the ICD-11 trait domains using a selection of 16 PID-5 facet scales. A consecutive study using exploratory structural equation modeling with this selection of PID-5 trait facets (Sellbom et al., 2019) found adequate model fit for a five-factor solution. Nevertheless, this approach omits essential trait facets that are required for the scoring of AMPD trait domains (e.g., separation insecurity), includes trait facets with high cross-loadings (e.g., hostility), and drops the entire trait domain of Psychoticism. The resulting measurement model is therefore not backward-compatible with the *DSM-5* trait model.

In both clinical and research settings, resources are often scarce and 220 item (PID-5) or even 100-item short-form (Maples et al., 2015) measures for maladaptive personality traits may be too lengthy for use in many circumstances, thus impeding their widespread adoption. Although a 25-item brief form exists for the PID-5 (PID-5-BF; American Psychiatric Association, 2013b), research on this brief form has revealed limitations. For instance, exploratory factor analysis assessing its structure yielded mixed results: The model fit was adequate, but some items had loadings below .30 and some items did not show the highest loading on their expected trait domain (Fossati et al., 2017). Another study using confirmatory factor analysis found acceptable but not optimal model fit for a five-factor solution (Anderson et al., 2018). Moreover, the PID-5-BF is not compatible with ICD-11 because it does not capture trait facets associated with Anankastia.

In this study, we used a novel but promising approach to item selection based on the ant colony optimization (ACO) meta-heuristic (Colorni et al., 1991; Leite et al., 2008) in order to derive a 34-item measure (i.e., the PID5BF+), which assesses 17 of the 25 facets of the PID-5 and covers all maladaptive trait domains of the *DSM-5* AMPD while being compatible with the ICD-11 maladaptive trait domains. Since the main difference between the two diagnostic models concerns the domains of Anankastia and Psychoticism, our resulting measurement model comprised the five *DSM-5* trait domains (Negative Affectivity, Detachment, Antagonism, Disinhibition, Psychoticism) plus the ICD-11 trait domain Anankastia, based on the ICD-11 “cross walk” for the *DSM-5* AMPD (Bach et al., 2017). The decision for this composite model and against the complete adoption of the algorithm by Bach et al (2017) was twofold: First, our goal was to build a measure compatible with both systems, which would not be the case if we omit trait facets and/or domains necessary for the *DSM-5* AMPD domain scoring algorithm. Second, a considerable amount of studies investigating the latent structure of the PID-5 including the metanalysis by Watters and Bagby (2018) could replicate the selection of 15 trait facets included in the AMPD scoring algorithm to have the highest specificity (high factor loadings and low cross-loadings) among the 25 PID-5 trait facets. Therefore, we aimed at a hierarchical measurement model based on the 15 facet traits included in the *DSM-5* AMPD scoring algorithm plus perseveration and rigid perfectionism as operationalization for ICD-11 Anankastia according to Bach et al. (2017).

We applied ACO to select a set of items that maximizes the reliability and validity of the trait domain and facet scales while providing a good model fit of the measurement model as well as cross-cultural measurement invariance. Our analyses were based on three different German- and English-speaking samples. We assessed criterion validity with measures of personality, maladaptive traits, and interpersonal problems and compared maladaptive personality trait profiles in clinical subgroups. In a final step, we validated the new measure in two German community samples.

## Method

We report how we determined our sample size, all data exclusions, all manipulations, and all measures in the study.

### Samples

Sample characteristics are summarized in Table 1. Our data for item selection comprised a total of 2,927 participants consisting of a clinical and a nonclinical German-speaking sample, and an English-speaking (the United States) nonclinical sample. The German clinical sample (Sample 1) took part in a study on *DSM-5* PD assessment in inpatient settings. Regarding this sample, clinical diagnoses according to ICD-10 were available. The clinical diagnoses were obtained by the reference therapist and the responsible physician or head physician. One major and up to six minor diagnoses could be coded, whereby in this sample a maximum of one PD diagnosis was assigned per patient. The nonclinical German-speaking sample (Sample 2) comprised participants who took part in a questionnaire study on personality and mental health at several universities in Germany, Austria, and the German-speaking part of Switzerland. The U.S. sample (Sample 3) consisted of undergraduates who completed a self-report questionnaire online for course credit. To validate the solutions of the item selection process, the three construction samples were split randomly in a training sample of 2,048 individuals and a test sample of 879 (30%) individuals. We decided for this ratio because a smaller test sample would not have had enough size to calculate a hierarchical latent model with 6 factors, 17 subfactors, and 34 indicators. The composition ratio of the total sample (23.3% Sample 1, 19.1% Sample 2, and 56.5% Sample 3) was kept the same in the training and test samples.

**Table 1.** Sample Characteristics.

No.	Type	Source	*N*	Age	Gender	Instruments
1	German clinical sample	Zimmermann, Masuhr et al. (2014)	683 (732)	*M* = 34.4 years; *SD* = 13; range = 18-70	63% Female	PID-5 Clinical diagnoses
2	German nonclinical sample	Zimmermann, Altenstein et al. (2014); Zimmermann et al. (2017)	560 (611)	*M* = 25.5 years; *SD* = 7.9; range = 18-61	83.6% Female	PID-5 MRS-30
3	U.S. nonclinical sample	A. G. C. Wright et al. (2013)	1,684 (1,860)	*M* = 18.8 years; *SD* = 1.75; range = 18-56	66% Female	PID-5 IIP-SC
4	German nonclinical sample	Zimmermann et al. (2020)	849 (924)	*M* = 42.6 years; *SD* = 16.1; range = 18-82	50% Female	PID5BF+
5	German nonclinical sample		493 (518)	*M* = 35.7 years; *SD* = 12.80; range 18-75	70.8% Female	PID5BF+ PiCD

*Note.* Sample sizes denote included and total number of participants in parentheses. PID-5 = Personality Inventory for *DSM-5*; MRS-30 = Minimum Redundancy Scale–30; IIP-SC = Inventory for Interpersonal Problems Short Circumplex; PID5BF+ = Personality Inventory for *DSM-5*, Brief Form Plus; PiCD = Personality Inventory for ICD-11.

An additional German-speaking nonclinical sample (Sample 4) was used for validating the factor structure of the final item set. The sample consisted of individuals who took part in a questionnaire study on personality pathology, with the age and gender distributions being roughly representative of the German population. Participants were recruited via survey provider clickworker.de offering monetary reimbursement. To ensure data integrity, bogus items were implemented in the survey and we only included participants who answered less than two out of four bogus items incorrectly and who took more than 8 minutes to complete the survey (more than 2.7 seconds per questionnaire item). Finally, to investigate the correlations between the PID5BF+ and the PiCD, we used a nonclinical sample (Sample 5) that took part in a further survey that was part of a Master Thesis.

All participants fulfilled our inclusion criteria of less than 10% missing items and scores within 2.5 standard deviations of the community average on measures of random or careless responding (average long string, Mahalanobis distance, even-odd-consistency).

### Measures

#### Personality Inventory for *DSM-5* (PID-5)

The PID-5 is a 220-item self-report questionnaire which was constructed to evaluate maladaptive personality traits in five main domains and 25 facets according to Criterion B of the AMPD included in the *DSM-5* (American Psychiatric Association, 2013a; Krueger et al., 2012; German version: Zimmermann, Altenstein et al., 2014). The PID-5 uses a 4-point response scale. The instrument has been extensively tested in clinical and nonclinical samples and has demonstrated adequate psychometric properties (Al-Dajani et al., 2016; Zimmermann et al., 2019). The PID-5 was applied in the construction samples, and internal consistencies of the scales were adequate to high (*Mdn* α = .86; range = .71-.95).

#### Inventory of Interpersonal Problems–Short Circumplex (IIP-SC)

The IIP-SC is a 32-item self-report questionnaire designed to assess difficulties in interpersonal relationships (Soldz et al., 1995) on a 5-point response scale. The total score represents the amount of an individual’s interpersonal difficulties in daily life. IIP total and subscale scores were shown to be substantially associated with pathological personality traits (A. G. C. Wright et al., 2012). The IIP-SC was assessed in Sample 3, and internal consistencies were acceptable (*Mdn* α = .79; range = .71-.88).

#### Minimum Redundancy Scales–30-Item Version (MRS-30)

The MRS-30 comprises 30 pairs of adjectives that were selected to assess the Big Five personality factors with as little semantic overlap as possible (Schallberger & Venetz, 1999). Adjective pairs are rated on a 6-point bipolar response scale. The MRS was assessed in Sample 2, and internal consistencies were high (*Mdn* α = .81; range = .78-.90).

#### Personality Inventory for ICD-11

The PiCD is a self-report measure developed by Oltmanns and Widiger (2018) to assess PDs according to the diagnostic criteria of the ICD-11. It comprises 60 items with a 5-point response scale, of which 12 items are assigned to each of the domains Negative Affective, Disinhibition, Detachment, Dissocial, and Anankastic with high internal consistencies (*Mdn* α = .88; range = .84 – .89). We applied the German translation of the PiCD (Zettl & Volkert, 2019) in Sample 5.

### Ant Colony Optimization Algorithms

The selection of items for the construction of a short questionnaire scale with good psychometric properties can be understood as a combinatorial problem. In our case, the selection of 34 items for 17 facets of six domains from the respective 141 original items of these scales in the PID-5 would result in 4,022,467,735,750,944,579,649,536 possible combinations. Testing all of these combinations for (e.g.) model fit would take thousands of years on an average computer. We therefore applied an algorithmic approach to the item selection procedure based on the ACO metaheuristic. The ACO (Colorni et al., 1991) method is very effective for item selection and improving model fit (e.g., Janssen et al., 2017) and was demonstrated to perform better than traditional item selection strategies (Schroeders et al., 2016) as well as other metaheuristics such as genetic algorithms (Olaru et al., 2015) in designing five-factor short-scale assessments for personality. ACO is based on the food foraging behavior of ants and uses virtual “pheromones” to increase the attractiveness of item choices that yield good psychometric properties. As it is a probabilistic algorithm, it not necessarily finds the optimal solution. The user should therefore compare solutions yielded by several runs of the same algorithm or algorithms with different parameters to gain confidence in the final solution.

### Model Specification

We chose the three PID-5 facet traits per trait domain that had the highest loadings and the lowest cross-loadings according to the meta-analysis by Waters and Bagby (2018), with the addition of “perseveration” and “rigid perfectionism” to assess the trait domain Anankastia, based on the *DSM-5* ICD-11 crosswalk recommendations provided by Bach et al. (2017). This resulted in a measurement model including the 15 facet traits necessary for the *DSM-5* AMPD maladaptive trait domain scoring algorithm plus Anankastia for compatibility with the ICD-11 maladaptive trait model. We therefore specified a higher order factor model with items loading on their corresponding first-order factor, that is, one of 17 PID-5 facet traits, which in turn loaded on one of their respective PID-5 trait domains, with the exception of “perseveration” and “rigid perfectionism,” which loaded on Anankastia. The model was identified by constraining all unstandardized first- and second-order loadings to 1, leading to an essential tau-equivalent model. As the aim of this study was to develop a short measure, we chose to set the number of items per first-order factor to 2, resulting in a total of 34 items.

### Item Selection Procedure

The item selection was conducted using two different ACO-based algorithms in multiple runs with the aim of selecting two items per facet resulting in a selection of 34 items from the item pool of 141 PID-5 items. The first algorithm was an adaptation of the *MAX–MIN* Ant System (Stützle & Hoos, 2000), which is available as a function within the R package “stuart” (Schultze, 2018). In this case, we used a combination of model fit criteria root mean square error of approximation (RMSEA), standardized root mean square residual (SRMR) and the comparative fit index (CFI) as well as the average of facet- and domain-specific reliability in terms of McDonald’s ω. The second algorithm differed slightly in terms of the calculation of the optimization criterion and the definition of the converging criteria. In line with Schroeders et al. (2016), the calculation of the optimization criterion was based on the model fit (defined by RMSEA and CFI), reliability of the scale (defined by McDonald’s ω), the unstandardized minimum first- and second-order factor loadings with the addition of the average correlation between short and original versions of the trait facet scales (see Supplemental File 1 [available online] for details on the two algorithms).

In both algorithms, model fit and consistency criteria were calculated based on polychoric correlations with a diagonally weighted least squares estimator. Previous research suggests that robust categorical least squares methodology performs better than maximum likelihood estimators on data with fewer than five answer categories (Li, 2016; Rhemtulla et al., 2012), which is the case with the PID-5. CFI and RMSEA computations were based on scaled χ^2^ values according to Satorra and Bentler (2001). Every algorithm was run three times on the training data set and the model fit in terms of RMSEA, SRMR, and CFI was assessed in the test data set. The best three solutions regarding these model fit indices were then chosen for comparison concerning their internal consistency. Facet-item constellations that were not replicated at least twice were identified. To find unequivocal solutions for these facets, we calculated model fits, factor loadings and reliabilities for every possible combination of items yielded by the best three models of the previous steps. This was done with the “brute-force” function of the R package stuart. The final solution then consisted of the items possessing best content validity (judged by their semantic content) and reliability, generated the best model fit and yielded no Heywood cases (negative latent variances) in the test data set. We chose to apply this twofold algorithmic procedure with multiple runs, different parameters, and semantic comparison of solutions in order to maximize the probability of finding a global rather than local optimal solution.

### Evaluation of Model Fit, Measurement Invariance, and Criterion Validity

To assess model fit of the best shortened questionnaire solution generated in the previous steps, we used the common standards (i.e., RMSEA < .05, SRMR < .07, CFI > .95; Hu & Bentler, 1999; Marsh et al., 2005) of fit index interpretation. In addition, to be able to compare the measurement model quality of the newly generated short questionnaire to the already established PID-5-BF, we also calculated model fit for the measurement model with 25 items and five domains (five items per trait domain) underlying the PID-5-BF.

To further investigate measurement invariance between German- and English-speaking samples, we computed CFI, RMSEA with 90% confidence interval (CI) and SRMR for increasing levels of restricted model parameters. As we are using diagonal weighted least squares method estimation on ordinal data, we implemented the following steps of increased parameter constriction in line with Wu and Estabrook (2016): Model 1: fixed factor loading to 1 for one item per facet and one facet per higher order factor and one invariant threshold per item or facet; Model 2: equal item thresholds and latent intercepts across groups; Model 3: equal item thresholds, intercepts, first- and second-order factor loadings across groups, and Model 4: Equal thresholds, intercepts, first- and second-order factor loadings and equal item residual variances across groups. To compare observed scale means between groups, invariant thresholds, factor loadings, and residual variances are necessary. To determine which level of measurement invariance is fulfilled by our final model, we then calculated differences in CFI, RMSEA, and SRMR for each level of measurement invariance. According to Putnick and Bornstein (2016), a difference <.01 for CFI and SRMR as well as overlapping 90% CIs for the RMSEA between subsequent levels of measurement invariance indicate acceptable relative fit.

To further evaluate the quality of the newly generated short PID-5 version as a standalone measure, we assessed model fit and reliability in a separate validation sample (Sample 4). To assess convergent and discriminant validity of the newly generated scales in relation to the original PID-5 scales in the construction sample, individual correlations were first transformed using the Fisher’s Z transformation, before being averaged and transformed back into Pearson correlations. We investigated criterion validity using the (Fisher’s Z transformed) correlations with Big Five traits, assessed with the MRS-30 (Sample 2), and with interpersonal distress, assessed with the IIP-SC (Sample 3). This enabled us to calculate CIs for correlation differences according to Zou (2007) to evaluate the differences in the correlations of shortened and full versions of the measures. To investigate the convergence with maladaptive traits as defined in the ICD-11, correlations between the PiCD and the newly generated standalone measure were investigated in Sample 5.

To evaluate and compare the ability of the newly generated measure to differentiate between patient groups with mild or more severe mental health disorders without PD diagnoses from patients with PD diagnoses, we compared group means for facet and domain trait scores between three patient groups in Sample 1 using Cohen’s *d* and CIs. We selected all patients from the clinical subsample with clinical diagnoses, who had either no PD but mental disorders from the internalizing spectrum (Conway et al., 2019), that is, from the ICD-10 chapters F32, F33, F34, F40, F41, F42, F43, F50, F51, F52, F53, or a diagnosis of borderline PD. We then compared the group of patients with only one internalizing diagnosis (but no PD diagnosis) to the group of patients with three or more diagnoses from the internalizing spectrum (but no PD diagnosis), and in turn compared the latter with the group of patients with a borderline PD diagnosis. This approach allowed us to distinctly investigate the ability of the newly generated measure to distinguish between (a) mild and more severe mental health conditions and (b) the presence or absence of PD. We chose borderline PD as this is the only categorical description of PD that will remain in the ICD-11 (as a “borderline pattern specifier”) and because borderline PD symptomatology seems to render the general dimension for personality pathology (Clark et al., 2018; Kernberg, 2004; Sharp et al., 2015). Furthermore, we assessed facet and domain score differences between the shortened and original PID-5 scales in these different patient groups using Cohen’s *d* and CIs for long and short scale means. We applied the classical calculation method for Cohen’s *d* (Cohen, 1988) both for differences between patient groups and within patient groups between the short and long versions of the scale to ensure comparability of these effect sizes according to Morris and DeShon (2002). Concerning the interpretation of effect sizes, we considered a Cohen’s *d* of 0.2 as small, 0.5 as moderate, and 0.8 as large. For correlation coefficients, we considered a Pearson’s *r* of .1 as small, .3 as moderate, and .5 as large.

## Results

### Model Fit and Latent Structure in the Construction Sample

The model fit of the finally selected 34 PID-5 items representing 17 trait facets and six trait domains with increasing levels of parameter restrictions is presented in Supplemental Table S1 (available online). The most restrictive measurement model with equal thresholds, intercepts, first- and second-order factor loadings and equal item residual variances across groups showed only minor decreases in the model fit indices in comparison with the least restrictive measurement model and could therefore be accepted (CFI = .942, RMSEA = .046, SRMR = .061). Notably, model fit of the PID5BF+ omitting the Anankastia domain (yielding the AMPD five factor model) was CFI = .95, RMSEA = .047, SRMR = .060, for the most restrictive measurement model.

In contrast, applying the same procedure to the measurement model of the PID-5-BF with five items per trait domain was problematic, as one of the items (PID166) had zero frequency in the highest answer category in Sample 2. We therefore assessed model fit separately in the three samples for the PID-5-BF model, yielding poor to acceptable model fit, with CFI = .886, RMSEA = .067, SRMR = .077 in Sample 1, CFI = .892, RMSEA = .068, SRMR = .078 in Sample 2, and CFI = .903, RMSEA = .073, SRMR = .071 in Sample 3.

**Figure 1.**
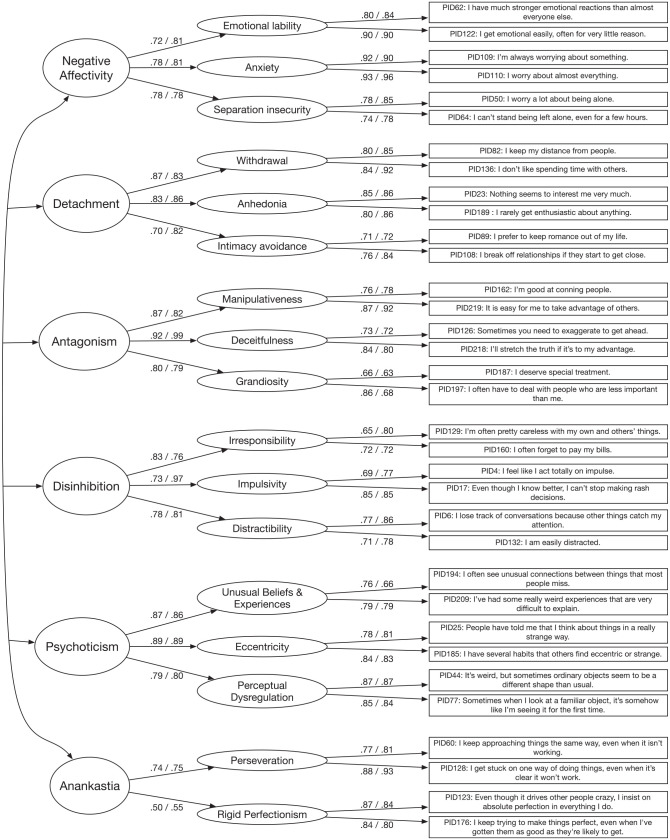
Latent measurement invariant model. *Note*. Depicted are standardized loadings, averaged over the three samples with *N* = 2,927 (left value) and standardized loadings in the separate validation sample with *n* = 849 (right value).

The final selection of items and the standardized factor loadings, averaged over the three samples, for trait facets and trait domains is depicted in Figure 1. In the following, numbers in brackets are average factor loadings calculated separately for the German clinical, the German nonclinical and the U.S.-English nonclinical samples. On the facet trait level, standardized item-factor loadings have an average of .80 [.79; .80; .80] with an average standardized error of .03 [.04; .04; .02], indicating good factor saturation. On the domain level, standardized facet-trait-domain factor loadings have an average of .79 [.78; .80; .79] with an average standardized error of .04 [.04; .05; .02], indicating good factor saturation of the latent trait domain factors, with the exception of rigid perfectionism, which showed a standardized average loading of .50 on Anankastia. Average manifest interdomain correlations ranged from .06 for Antagonism and Negative Affectivity to .48 for Anankastia and Negative Affectivity with an average of .32.

### Model Fit and Latent Structure in a Separate Validation Sample

Model fit of the 34-item hierarchical PID5BF+ model in Sample 4 was good (CFI = .941, RMSEA = .055, SRMR = .059). Yet the estimation based on polychoric correlations with a diagonally weighted least squares estimator resulted in two Heywood cases hindering the interpretation of the latent model: a negative variance for the Antagonism facet deceitfulness and a very high latent correlation between the domains Anankastia and Negative Affectivity. We therefore estimated the PID5BF+ model in the validation sample using Bayesian CFA with ordered indicators and continuous latent variables in M*plus* 8.0 (Muthén et al., 2017). Average latent item-facet as well as facet-domain loadings were .82, with a standardized error of .03, indicating a saturated latent factor structure of the newly generated short PID-5 measure assessed in a separate validation sample (see Figure 1 for factor loadings). As Sample 4 was roughly representative of the German population in terms of age and gender, we generated preliminary norm values for the PID5BF+, which are available in the Supplemental File 2 (available online).

### Reliability

For the assessment of reliability of the PID5BF+ scales in both construction and validation samples, we calculated McDonald’s ω for facet and domain scales (see Table 2) as a measure of model-based reliability (McDonald, 1970, 1999). All domain reliabilities were satisfactory, with the exception of Anankastia in the two nonclinical samples. All facet reliabilities were satisfactory, with anxiety having the highest values and irresponsibility having the lowest. Average within-domain correlations of raw facet scores were .45 for Negative Affectivity, .48 for Detachment, .49 for Antagonism, .37 for Disinhibition, .48 for Psychoticism, and .25 for Anankastia.

**Table 2.** McDonald’s ω for Trait Facets and Domains of the Reduced PID-5 Item Set.

	German clinical sample (1)	German nonclinical sample (2)	U.S. nonclinical sample (3)	German nonclinical validation sample (4)
Negative Affectivity	.77	.83	.82	.84
Emotional Lability	.85	.86	.80	.86
Anxiety	.92	.93	.93	.93
Separation Insecurity	.79	.74	.65	.80
Detachment	.81	.85	.86	.88
Withdrawal	.78	.86	.78	.88
Anhedonia	.78	.82	.83	.85
Intimacy Avoidance	.62	.76	.72	.75
Antagonism	.92	.88	.89	.90
Manipulativeness	.80	.79	.82	.84
Deceitfulness	.79	.72	.80	.73
Grandiosity	.75	.75	.71	.60
Disinhibition	.77	.86	.84	.88
Irresponsibility	.62	.64	.67	.73
Impulsivity	.70	.73	.82	.79
Distractibility	.72	.69	.71	.80
Psychoticism	.88	.88	.90	.89
Unusual Beliefs and Experiences	.73	.78	.74	.70
Eccentricity	.75	.83	.80	.80
Perceptual Dysregulation	.86	.87	.83	.85
Anankastia	.64	.53	.52	.61
Perseveration	.79	.82	.84	.86
Rigid Perfectionism	.85	.88	.83	.80
Mean (trait facets)	.77	.79	.78	.80
Mean (trait domains)	.80	.81	.81	.83

*Note*. PID-5 = Personality Inventory for *DSM-5*.

### Convergent and Discriminant Validity Regarding the Original Version of the PID-5

**Figure 2.**
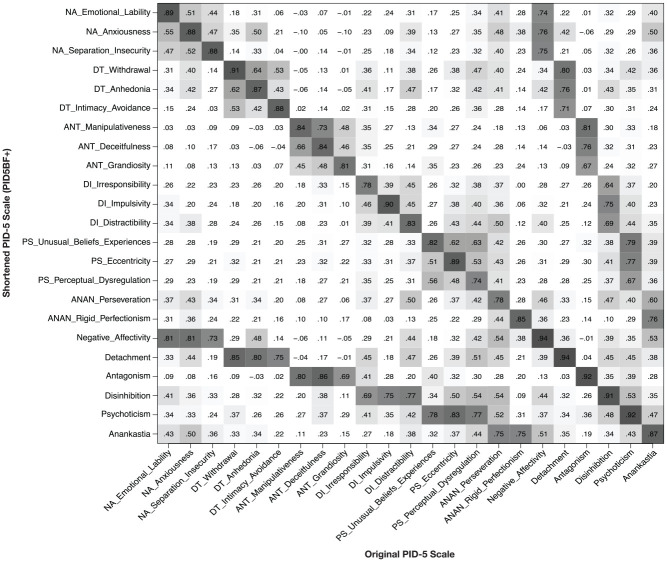
Pearson correlations between shortened and original *PID-5* scales calculated with data from all samples (*N* = 2,927). *Note.* Correlations >.29 are marked in gray with increasing darkness depending on the extent of the correlation. NA = Negative Affectivity; DT = Detachment; ANT = Antagonism; DI = Disinhibition; PS = Perceptual Dysregulation; ANAN = Anankastia; PID-5 = Personality Inventory for *DSM-5*.

Convergent validity correlations between the shortened and the original versions of the PID-5 trait facet and domain scales are depicted in Figure 2. In the following, numbers in brackets are average convergent correlations calculated separately for the German clinical, the German nonclinical and the U.S.-English nonclinical samples. At the facet level, the average convergent correlation between short and long versions of the scales was .85 [.84; .85; .85], ranging from .74 [.75; .73; .73] for perceptual dysregulation to .91 [.89; .93; .90] for withdrawal. Convergent correlations on the domain level ranged from .87 [.86; .87; .86] for Anankastia to .94 [.93; .95; .94] for Negative Affectivity, with a mean of .92 [.91; .92; .92].

We further examined discriminant validity correlations between the PID5BF+ and original PID-5 facet and domain scores (see Figure 2). As above, plain numbers are correlation coefficients over the total construction sample of *N* = 2,927, and correlation coefficients in brackets are calculated separately for the German clinical, the German nonclinical and the U.S.-English nonclinical samples. Concerning facet trait scores, the average discriminant correlation between short and long versions of the scales was .23 [.19; .23; .25], ranging from −.10 [−.10; −.13; −.04] for anxiousness and manipulativeness to .50 [.44; .53; .48] for distractibility and perseveration. Average discriminant correlations between short and long versions of the trait domain scores were .35 [.29; .34; .36], with a range from −.01 [−.03; .01; .10] for Antagonism and Negative Affectivity to .53 [.47; .50; .51] for Disinhibition and Psychoticism.

### Criterion Validity

Table 3 shows the correlation differences of the full and shortened PID-5 scales with the five MRS-30 personality trait domains as well as interpersonal distress measured by the IIP-SC. In the following, numbers in brackets are averaged correlation coefficients for the short and the original versions of the respective PID-5 scales mentioned. Concerning the Big Five personality traits, Negative Affectivity domain and facet trait scores had moderate to strong associations with Neuroticism [.64; .70], Detachment domain and facet trait scores had moderate associations with Extraversion [−.62; −.68] and Neuroticism [.39; .49], Disinhibition domain and trait facet scores had moderate associations with Conscientiousness [−.43; −.48], and Antagonism domain and trait facet scores showed rather weak associations with Agreeableness [−.20; −.19]. Psychoticism domain and facet scores were weakly correlated with Openness [.16; .15]. While Anankastia domain and trait facet scores were moderately and uniformly correlated with Neuroticism [.40; .46], only the rigid perfectionism trait facet score had weak to moderate associations with Conscientiousness [.32; .38]. Interpersonal distress was moderately associated with all domain and facet trait scores [.38; .40], with the exception of rigid perfectionism, impulsivity, and Antagonism scores, for which correlations were only weak.

**Table 3.** Pearson Correlations Between Interpersonal Distress (Inventory of Interpersonal Problems; IIP-SC; U.S. Sample, *n* = 1,653); Neuroticism, Extraversion, Agreeableness, Conscientiousness (Minimum Redundancy Scale; MRS-30; German Nonclinical Sample, *n* = 683) With Shortened and Original PID-5 Scales.

Trait	Scale [CI]	IIP interpersonal distress	MRS Neuroticism	MRS Extraversion	MRS Agreeableness	MRS Conscientiousness	MRS Openness
NA Emotional Lability	Short	.34***	.60***				
	long	.44***	.66***				
	CI diff.	**[−.13, −.09]**	[−.09, −.027]				
NA Anxiousness	short	.46***	.66***	−.20**			−.15
	long	.53***	.76***	−.26***			−.18**
	CI diff.	[−.097, −.056]	**[−.129, −.071]**	[.022, .098]			[−.006, .07]
NA Separation Insecurity	short	.37***	.55***		−.13		
	long	.40***	.59***		−.17*		
	CI diff.	[−.055, −.01]	[−.077, −.007]		[−.005, .079]		
DT Withdrawal	short	.46***	.33***	−.69***	−.20***		
	long	.50***	.34***	−.74***	−.20***		
	CI diff.	[−.062, −.022]	[−.042, .026]	[.029, .081]	[−.038, .033]		
DT Anhedonia	short	.47***	.45***	−.43***	−.17*	−.16*	−.27***
	long	.54***	.67***	−.54***	−.18**	−.09	−.29***
	CI diff.	[−.093, −.051]	**[−.272, −.182]**	**[.063, .153]**	[−.045, .055]	[−.124, −.024]	[−.034, .064]
DT Intimacy Avoidance	short	.32***	−.15*	−.35***	−.18**		
	long	.35***	.20***	−.44***	−.15*		
	CI diff.	[−.046, −.002]	[−.099, −.013]	**[.043, .124]**	[−.075, .011]		
ANT Manipulativeness	short	.22***	−.16*	.03	−.24***	−.16*	.06
	long	.12***	−.26***	.24***	−.14	−.05	.17*
	CI diff.	[.068, .12]	[.047, .17]	[−.275, −.151]	[−.162, −.038]	[−.175, −.05]	[−.172, −.047]
ANT Deceitfulness	short	.28***			−.17*	−.19**	
	long	.35***			−.27***	−.19**	
	CI diff.	[−.091, −.039]			[.044, .155]	[−.053, .058]	
ANT Grandiosity	short	.24***	.02		−.15*		.1
	long	.1***	−.2***		−.14		.2**
	CI diff.	[.108, .166]	[.157, .276]		[−.075, .045]		[−.158, −.037]
DI Irresponsibility	short	.36***	.19**		−.16*	−.52***	
	long	.44***	.23***		−.21***	−.49***	
	CI diff.	**[−.104, −.048]**	[−.098, .026]		[−.007, .117]	[−.088, .02]	
DI Impulsivity	short	.25***	.17*	.18**	−.2***	−.31***	
	long	.23***	.12	.29***	−.24***	−.39***	
	CI diff.	[−.003, .038]	[.01, .094]	[−.156, −.073]	[.001, .084]	**[.042, .122]**	
DI Distractibility	short	.37***	.34***	−.01		−.37***	
	long	.48***	.44***	−.13		−.46***	
	CI diff.	**[−.138, −.087]**	**[−.154, −.055]**	[.061, .168]		**[.046, .144]**	
PS Unusual Beliefs Experiences	short	.35***	.17*	−.2**		−.11	.17*
	long	.32***	.05	−.17**		−.09	.23***
	CI diff.	[.009, .065]	[.073, .166]	[−.072, .02]		[−.061, .032]	[−.103, .011]
PS Eccentricity	short	.33***	.22***	−.3***	−.28***	−.2***	
	long	.41***	.27***	−.36***	−.22***	−.22***	
	CI diff.	**[−.101, −.061]**	[−.096, −.007]	[.01, .097]	[−.104, .014]	[−.021, .069]	
PS Perceptual Dysregulation	short	.27***	**.12**	−.14		−.12	.17*
	long	.5***	.41***	−.26***		−.16*	.09
	CI diff.	**[−.263, −.198]**	**[−.363, −.229]**	[.046, .183]		[−.034, .105]	[.012, .152]
ANAN Perseveration	short	.46***	.39***	−.23***		−.17**	
	long	.56***	.52***	−.29***		−.17*	
	CI diff.	**[−.131, −.078]**	**[−.183, −.073]**	[−.004, .115]		[−.07, .052]	
ANAN Rigid Perfectionism	short	.24***	.34***	−.23***		.32***	
	long	.26***	.39***	−.28***		.38***	
	CI diff.	[−.044, .006]	[−.09, −.002]	[.011, .102]		**[−.104, −.015]**	
Negative Affectivity	short	.49***	.75***				
	long	.55***	.79***				
	CI diff.	[−.076, −.046]	[−.059, −.02]				
Detachment	short	.54***	.39***	−.62***	−.23***		−.19**
	long	.56***	.49***	−.68***	−.21***		−.21***
	CI diff.	[−.041, −.012]	**[−.128, −.067]**	[.038, .09]	[−.056, .008]		[−.017, .048]
Antagonism	short	.3***	−.06		−.24***	−.16*	.07
	long	.22***	−.19**		−.22***	−.1	.17*
	CI diff.	**[.064, .101]**	[.08, .172]		[−.063, .027]	[−.113, −.022]	[−.151, .06]
Disinhibition	short	.42***	.31***		−.17**	−.52***	
	long	.46***	.35***		−.21***	−.57***	
	CI diff.	[−.053, −.018]	[−.076, −.001]		[−.001, .077]	[.012, .079]	
Psychoticism	short	.4***	.21***	−.27***	−.18**	−.18**	.19**
	long	.47***	.28***	−.31***	−.16*	−.19**	.17*
	CI diff.	[−.09, −.055]	[−.104, −.034]	[.005, .075]	[−.051, .021]	[−.027, .044]	[−.01, .061]
Anankastia	short	.43***	.46***	−.29***		.14	
	long	.47***	.51***	−.33***		.15*	
	CI diff.	[−.058, −.013]	[−.096, −.017]	[−.003, .082]		[−.058, .031]	

*Note*. The upper value in each cell is the correlation with the short scale, the lower value is the correlation with the original scale. Values in brackets are 95% CIs for the mean differences of the correlations, calculated according to Zou (2007). Correlations >.3 with correlation difference CIs containing .1 or −.1 are marked in bold. NA = Negative Affectivity, DT = Detachment, ANT = Antagonism, DI = Disinhibition, PS = Psychoticism, ANAN = Anankastia; PID-5 = Personality Inventory for *DSM-5*; CI = confidence interval; MRS = Minimum Redundancy Scale.

To evaluate differences in the correlations of the short and original versions of the PID-5 scales with MRS-30 and IIP scales, we investigated all correlations ≥.30, that is, those that were at least moderate. The overall average difference of at least moderate correlations between short and long versions of the PID-5 with MRS and IIP scores was .07 and all correlations were in the same direction. On the domain level, differences in at least moderate correlations that included |.10| in their confidence interval were found for Antagonism and interpersonal distress (.30 vs. .22) and for Detachment and Neuroticism (.39 vs. .49). Concerning the facet trait scales, short and long scale versions for Anhedonia (.45 vs. .67 with Neuroticism; −.43 vs. −.54 with Extraversion), Distractability (.37 vs. .48 with interpersonal distress; .34 vs. .44 with Neuroticism), Perceptual Dysregulation (.27 vs. .50 with interpersonal distress; .12 vs. .41 with Neuroticism) and Perseveration (.46 vs. .56 with interpersonal distress; .39 vs. .52 with Neuroticism) had the most remarkable correlation differences.

Table 4 shows the correlations between the PID5BF+ domain and facet scores and PiCD trait domains in Sample 5. Beside Psychoticism, all PID5BF+ trait domains showed moderate to strong correlations with the expected PiCD trait domains with Negative Affectivity domains showing the largest (*r* = .81) and Anankastic (PiCD) and Anankastia (PID5BF+) showing the lowest (*r* = .50) convergence. A negative correlation of -.20 was found between PID5BF+ Disinhibition and PiCD Anankastic. All PID5BF+ trait facets showed the highest correlation with the expected PiCD trait domain with the exception of perseveration, which mainly correlated with the Negative Affective domain. However, the Anankastia facet rigid perfectionism (PID5BF+) showed a high correlation with PiCD Anankastic (.58). The PID5BF+ trait domain Psychoticism and its trait facets showed moderate correlations with PiCD trait domains Dissocial and Disinhibition.

**Table 4.** Pearson Correlations Between the Five Maladaptive Personality Scales Negative Affective, Disinhibition, Detachment, Dissocial, Anankastic (Personality Inventory for ICD-11 [PiCD]; German Validation Sample, *n* = 493) With PID5BF+ Scales.

Trait	PiCD				
	Negative Affective	Detachment	Dissocial	Disinhibition	Anankastic
NA Emotional Lability	**.60**	−.03	.12	.24	.13
NA Anxiousness	**.77**	.33	.12	.13	.41
NA Separation Insecurity	**.56**	.16	.18	.25	.22
DT Withdrawal	.38	**.72**	.23	.13	.32
DT Anhedonia	.32	**.56**	.21	.14	.24
DT Intimacy Avoidance	.30	**.49**	.29	.17	.16
ANT Manipulativeness	.08	.08	**.59**	.26	−.02
ANT Deceitfulness	.26	.19	**.47**	.29	.06
ANT Grandiosity	.19	.11	**.46**	.24	.10
DI Irresponsibility	.11	.09	.24	**.58**	−.22
DI Impulsivity	.36	.06	.38	**.64**	−.19
DI Distractibility	.35	.14	.21	**.49**	−.06
ANAN Perseveration	**.41**	.30	.20	.28	.14
ANAN Rigid Perfectionism	.38	.26	.24	−.03	**.58**
PS Unusual Beliefs and Experiences	.24	.16	**.29**	.28	.06
PS Eccentricity	.34	.35	**.44**	.36	.12
PS Perceptual Dysregulation	.18	.12	.27	**.30**	−.00
Negative Affectivity	**.81**	.20	.17	.24	.32
Detachment	.43	**.76**	.31	.19	.31
Antagonism	.23	.17	**.65**	.34	.06
Disinhibition	.37	.13	.37	**.75**	−.20
Anankastia	**.50**	.35	.28	.13	**.50**
Psychoticism	.32	.28	**.42**	.39	.07

*Note*. All *r* > |.08| are significant at *p* < .05. Values in bold depict the highest correlation in the row. NA = Negative Affectivity; DT = Detachment; ANT = Antagonism; DI = Disinhibition; PS = Psychoticism; ANAN = Anankastia.

### Differences in PID-5 and PID5BF+ Between Averaged Trait Profiles in Clinical Subgroups

**Figure 3.**
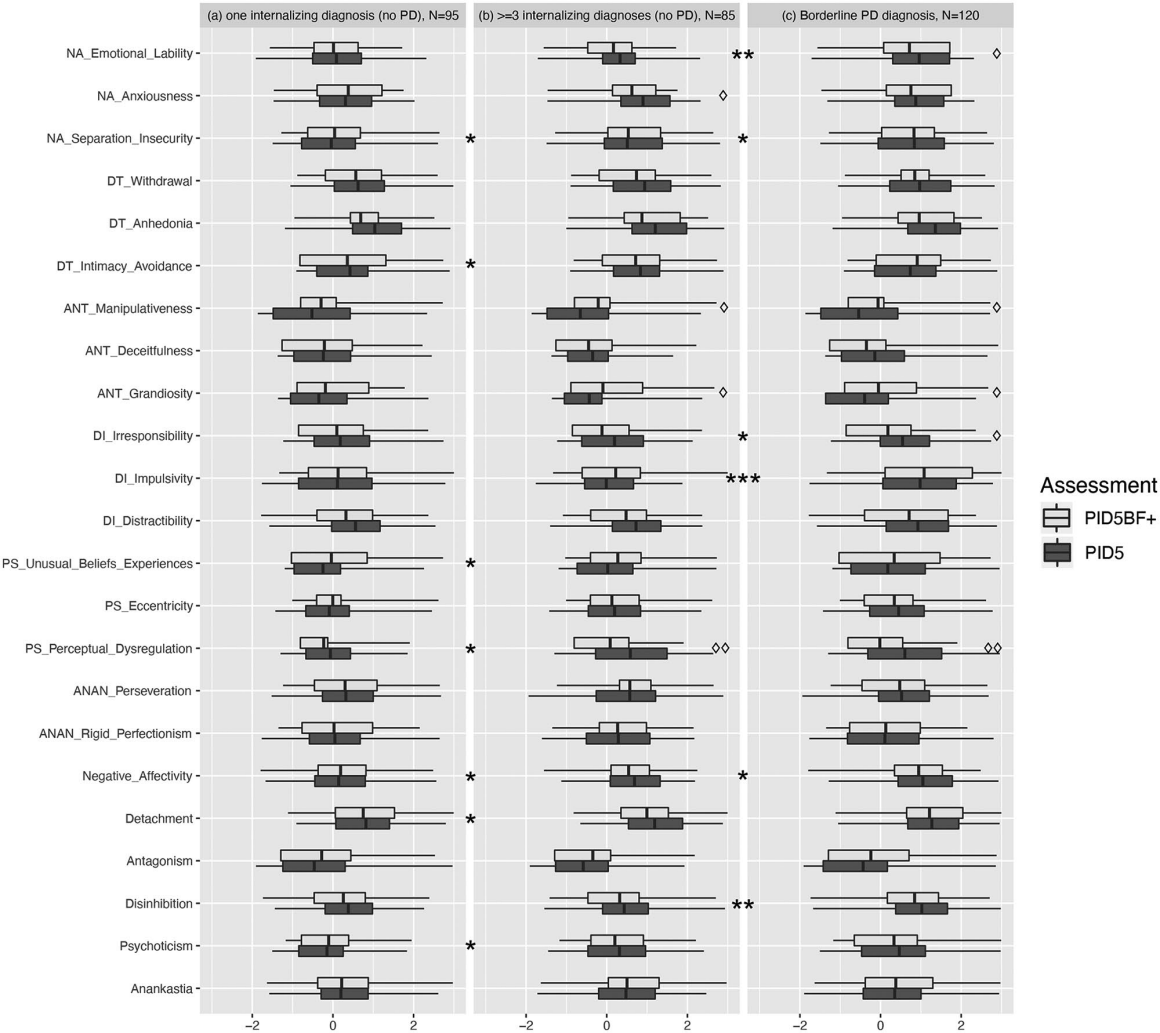
Distribution (25% and 75% quartiles) and average scores of short and original versions of PID-5 scales in psychiatric inpatients with one (a) three or more (b) diagnoses from the internalizing spectrum (F32, F33, F34, F40, F41, F42, F43, F50, F51, F52, F53, ICD-10) or borderline PD diagnosis (c). *Note*. All scores are *z*-standardized in relation to the German nonclinical sample. Asterisks denote significant (i.e., not containing zero within the confidence interval) between group difference effects (Cohens’ *d*) on PID5BF+ scales, deltoids denote significant difference effects between PID5BF+ and PID-5 scales, * or ◊ = 0.2 < *d* <.5, ** or ◊◊ = .5 < *d* < .8, *** or ◊◊◊ = *d* > .8. PID-5 = Personality Inventory for *DSM-5*.

Figure 3 presents *z*-standardized scores (in comparison with Sample 2) for facet and domain trait scales of the short and original PID-5 scales in three patient groups: (a) patients with one diagnosis from the internalizing spectrum but without PD, (b) patients with ≥3 diagnoses from the internalizing spectrum but without PD, and (c) patients with Borderline PD diagnosis. Significant differences (i.e., Cohen’s *d* with CIs not containing zero) between averaged scores of short and original scale versions were found for manipulativeness (higher score on the short scale in patient Groups (b) *d* = 0.48, CI [0.17, 0.79] and (c) *d* = 0.35, CI [0.10, 0.61]); grandiosity (higher score on the short scale in Groups (b) *d* = 0.37, CI [0.06, 0.59] and (c) *d* = 0.33, CI [0.07, 0.59]); irresponsibility (lower score on the short scale in Group (c) *d* = 0.32, CI [0.06, 0.58]); emotional lability (lower score on the short scale in Group (c) *d* = 0.26, CI [0.01, 0.52]); anxiousness (lower score on the short scale in Group (b) *d* = 0.33, CI [0.02, 0.63]) and perceptual dysregulation (lower score on the short scale in Group (b) *d* = 0.54, CI [0.23, 0.85] and Group (c) *d* = 0.52, CI [0.26, 0.78]).

Concerning between-group differences captured by the short scale version, differences with CIs not containing zero emerged between the groups defined by single versus multiple comorbid diagnoses from the internalizing spectrum for Negative Affectivity (*d* = 0.41, CI [0.11, 0.70]); Detachment (*d* = 0.38, CI [0.09, 0.68]); and Psychoticism (*d* = 0.36, CI [0.06, 0.65]) on the domain trait level and for separation insecurity (*d* = 0.53, CI [0.12, 0.94]); intimacy avoidance (*d* = 0.39, CI [0.09, 0.69]); unusual beliefs and experiences (*d* = 0.31, CI [0.02, 0.61]); and perceptual dysregulation (*d* = 0.37, CI [0.07, 0.66]) on the facet trait level. Significant PID5BF+ scale difference effects between the groups defined by multiple comorbid internalizing diagnoses vs. borderline PD diagnoses were found for Negative Affectivity (*d* = 0.46, CI [0.17, 0.75]) and Disinhibition (*d* = 0.60, CI [0.31, 0.89]) on the domain trait level and for emotional lability (*d* = 0.61, CI [0.31, 0.90]; irresponsibility (*d* = 0.30, CI [0.01, 0.58]); and impulsivity (*d* = 0.81, CI [0.50, 1.11] on the facet trait level.

The between-group comparison of the average score of all 17 maladaptive trait facets with single versus multiple comorbid internalizing diagnoses revealed a small to medium difference of *d* = 0.39, CI [0.09, 0.69], which was substantially higher for the comparison between the groups with single internalizing diagnoses versus borderline PD diagnoses (*d* = 0.74, CI [0.45, 1.0]). The comparison of the average facet score between the groups with multiple comorbid internalizing diagnoses and borderline PD diagnosis resulted in a (nonsignificant) small to medium difference (*d* = 0.42, CI [−0.05, 0.90]).

## Discussion

The shift from categorical to dimensional models and assessments of personality pathology in the *DSM-5* and in the ICD-11 represents an important step toward an empirically grounded nosology (Hengartner et al., 2018; Hopwood et al., 2018; Tyrer et al., 2018). Furthermore, maladaptive personality traits seem to represent predictive and transdiagnostic factors for general psychopathology (Bach & Bernstein, 2018; Hopwood, 2018b; A. G. C. Wright & Simms, 2015) as reflected by their prominent inclusion in emerging dimensional models of general psychopathology (Kotov et al., 2017; Widiger et al., 2019). However, this paradigm shift also poses a challenge regarding dissemination and application in standard health care situations. Consequently, brief but reliable and valid measures to assess personality pathology according to the new models are urgently needed. To this aim, the present study used ant colony optimization algorithms to generate a maximally valid and reliable 34-item measure for *DSM-5* maladaptive personality traits that is also compatible with the ICD-11 model.

### Internal Consistency and Latent Structure

The average model-based reliability (McDonald’s ω) of .81 for the domain trait scores and .79 for the facet trait scores demonstrated good internal consistency in all samples including the separate validation samples. These average values concerning model-based reliability are comparable to previous findings (Quilty et al., 2013) on the 220-item PID-5 version, implying good reliability of the PID5BF+ despite the substantial reduction of the number of items. An exception lies in the domain of Anankastia with an average reliability of .58. Considering the good reliability of the underlying facet traits perseveration and rigid perfectionism, this finding points to the notion that perseveration and rigid perfectionism may, though sharing common variance, partly be grounded in different constructs. This interpretation is also supported by the comparably low intercorrelation of .25 between scores of perseveration and rigid perfectionism. Furthermore, in recent meta-analyses (Somma et al., 2019; Watters & Bagby, 2018), rigid perfectionism showed a significant (inverse) loading on Disinhibition while perseveration did not, and both trait facets were consistently loading on Negative Affectivity. The latter may be an explanation for the relatively large correlation of .48 between scores of Negative Affectivity and Anankastia.

Nevertheless, model fit parameters calculated with a cross-culturally measurement invariant measurement model over three samples with 2,927 participants showed good model fit, which was considerably better than the model fit for the 25 items included in the PID-5-BF. An explanation for this difference may be the superiority of the ACO algorithm for selecting cross-cultural invariant item sets compared with traditional item selection strategies (Olaru & Danner, 2020). The latent hierarchical model with 17 first-order and 6 second-order factors showed average factor loadings of .80 in the construction samples and .82 in the separate validation sample. Despite the above mentioned limitation concerning Anankastia, the homogeneous distribution of factor loadings as well as the good model fit of the cross-culturally measurement invariant model allow for the comparison of sum or mean scores of the PID5BF+ between groups and individuals.

### Convergent and Discriminant Validity

Since the publication of the PID-5 in 2012, a huge amount of research supporting its validity has accumulated (Zimmermann et al., 2019). Scores from the PID5BF+ scales demonstrated strong convergent validity with scores from the original PID-5 scales, with the mean convergent validity correlation lying at .92 on the domain level and .85 on the trait facet level. All correlations between shortened and original scales were strong and in the expected direction. However, these correlations need to be interpreted with caution, as the PID5BF+ items are contained within the PID-5. This leads to an inflation of correlation estimates as partly, the same item data were entered in the correlation calculations since the short and long versions of the measure were not assessed separately. Nevertheless, taken together with the above-described good internal consistency and internal structure of the newly developed short measure, the strong convergent correlations with the original scales suggest a good usability of the PID5BF+ as a diagnostic measure for maladaptive personality traits according to *DSM-5*.

In contrast to previous findings regarding the discriminant validity of the PID-5 with average scale intercorrelations of .49 for the domain scales and .36 for the trait facet scales (Crego, Gore, et al., 2015), the average discriminant correlation of the PID5BF+ was .34 for the domain scales and .23 for the trait facet scales. The lower discriminant correlation, that is, the higher discriminant validity of the PID5BF+ is probably due to the exclusion of the interstitial trait facets of the PID-5, which load on more than one trait domain. These interstitial facets are also omitted in the official scoring algorithm for the PID-5 trait domains. The moderate correlation between perseveration and distractibility, which was previously found to be even higher for the original PID-5 scales (Crego et al., 2015) may be explained by a common etiological processes as both facets are indicative for attention-deficit/hyperactivity disorder (Smith & Samuel, 2017) and tend to merge in the same factor in some exploratory factor analyses (Bach et al., 2017; Zimmermann, Altenstein, et al., 2014).

### Criterion Validity

As probably the most important indicator among the various validity estimates, we investigated criterion validity by means of correlations of the PID5BF+ scores with ICD-11 maladaptive trait domains (PiCD), Big Five personality traits (MRS-30) as well as interpersonal distress (IIP-SC), and by investigating its ability to differentiate between patient groups using clinical diagnoses. All PID5BF+ trait domains showed significant correlations to the expected PiCD trait domains with PID5BF+ and PiCD Negative Affectivity, Disinhibition and Detachment domains showing strong correlations, Antagonism/Dissociality domains showing a moderate to strong, and Anankastia domains showing only a moderate correlation between the two measures. While these findings indicate a considerable overlap of the ICD-11/PID5BF+ maladaptive trait domains, the comparably lower intercorrelation of the two Anankastia operationalizations may be attributed to the rather low correlation of the PID5BF+ trait facet perseveration with PiCD Anankastia compared to rigid perfectionism. In contrast, perseveration showed a moderate correlation to PiCD Negative Affective, which may explain the moderate correlation between PID5BF+ Anankastia and PiCD Negative Affective trait domains.

All significant correlations with the Big Five personality traits were in the same direction as with the original scales. Correlation strength and direction of the trait facets was in line with previous findings from this sample (Zimmermann, Altenstein, et al., 2014), that is, anxiousness, emotional lability and separation insecurity had the highest associations with neuroticism, withdrawal, intimacy avoidance, and anhedonia had the highest (inverse) associations with extraversion, with anhedonia also being correlated with neuroticism; irresponsibility, impulsivity, and distractibility had the highest (inverse) associations with conscientiousness; and manipulativeness, deceitfulness, and grandiosity had weak associations with agreeableness. The newly constructed Anankastia domain showed only very low correlations to conscientiousness while its facet rigid perfectionism showed notable higher correlations than perseveration. This is in line with previous findings concerning the differential association of perseveration and rigid perfectionism with Big Five conscientiousness (Watson et al., 2013). Furthermore, both perseveration and rigid perfectionism were significantly associated with Neuroticism, which is in line with previous findings showing substantive loadings of these two PID-5 trait facets on Big Five Neuroticism (Suzuki et al., 2015).

Correlation coefficients of the PID5BF+ with Big Five traits were comparable to the findings of Al-Dajani et al. (2016) with the exception of Agreeableness and Antagonism (−.20 vs. −.62). The notable difference concerning Antagonism might stem from different domain scoring algorithms in previous studies. For instance, some studies used all trait facets to calculate domain scores, while others used the domain scoring approach proposed on the *DSM-5* website based on the three highest loadings facets of each domain. The latter approach, which is also the case with the PID5BF+ domains, leads to the exclusion of the trait facets callousness and hostility, which have the highest correlations with agreeableness among the Antagonism traits (e.g., Watson et al., 2013). A further source for the low correlation between Antagonism and agreeableness may be the implementation of agreeableness in the MRS-30, which might slightly differ from other Big Five measures. The weak association between MRS-30 Openness and both short and long versions of Psychoticism domain and facet scores in turn is in line with previous findings concerning weak or inconsistent associations between Big Five Openness and *DSM-5* AMPD Psychoticism (e.g., Suzuki et al., 2017; Widiger & Crego, 2019).

Correlations of PID5BF+ domain scores with interpersonal problems were also in a comparable range to previous findings by A. G. C. Wright et al. (2012). Again, the notable differences might stem from the domain scoring algorithm in A. G. C. Wright et al. (2012), which used all 25 trait facets. However, in a recent study comparing the domain scoring methods for the PID-5, Watters, Sellbom, et al. (2019) recommended the domain scoring algorithm we employed in this study to construct the PID5BF+ using the three highest loading facets. Furthermore, the absolute average difference between all correlations of PID-5 and PID5BF+ facet and domain scores with Big Five traits and interpersonal distress was .07, which corroborates the differing domain scoring algorithms in previous studies as the main source of the above-reported deviations.

The most notable differences concerning correlations to external measures and mean scores between the long and short scale version was found for perceptual dysregulation. This scale also showed the most remarkable differences in mean scores between the short and long versions in the profile comparisons between different patient groups. Thus, the scale and construct of perceptual dysregulation might be vulnerable to item reductions. This interpretation is supported by the findings of Maples et al. (2015), where perceptual dysregulation showed the highest drop among all PID-5 trait facets in terms of reliability and convergent correlations after reducing the number of items. One explanation for this could be that PID-5 perceptual dysregulation both integrates features of dissociative disorders (e.g., “People often talk about me doing things I don’t remember at all”) and features from the psychosis spectrum (e.g., “Sometimes I think someone else is removing thoughts from my head”) that may not be completely captured in the PID5BF+ after the reduction to just two items. However, a correlation of .92 between PID5BF+ and PID-5 Psychoticism scores suggested good agreement for the superordinate trait domain.

A more general discussion concerns the validity of the trait domain of Psychoticism itself as it showed moderate positive correlations with almost all external indicators of personality problems including 4 of the 5 PiCD domains, neuroticism, and interpersonal distress. While there is an ongoing debate whether psychotic symptoms such as hallucination and delusion and schizotypal personality traits belong to the same construct (see, e.g., Widiger & Crego, 2019), empirical evidence suggests that hallucinations, delusions and unusual thought content are associated with more severe cases of PD, at least concerning Borderline PD (Niemantsverdriet et al., 2017). A theoretical explanation for this association can be found in object relations theory and psychodynamic models of personality organization which assume that higher PD severity may involve psychotic-like experiences due to a highly vulnerable inner structure (Caligor et al., 2018; Kernberg, 2004). Furthermore, *DSM-5* Psychoticism seems to be predictive for other mental health conditions such as psychosis spectrum disorders (Bastiaens et al., 2019; Longenecker et al., 2020) and posttraumatic stress disorder (PTSD; James et al., 2015; Waszczuk et al., 2018) and may therefore play an important role beyond PD such as in the new ICD-11 diagnosis “complex PTSD.” One interpretation of these findings concerning the associations of *DSM-5* Psychoticism with psychopathological comorbidity and PD severity may be that *DSM-5* Psychoticism is an especially useful indicator of a vulnerable personality structure, which would be in line with thought disorder symptoms found to be at the “pinnacle” of general psychopathology liability conceptualizations such as the p-factor (Caspi et al., 2014). On the other hand, these findings concerning the centrality of thought disorder symptoms for general psychopathology may be unstable (Levin-Aspenson et al., 2020) or constitute statistical artifacts (Heinrich et al., 2020). Thus, broad associations of Psychoticism with a range of other mental disorders as described above or multiple PDs such as found in Watters, Bagby, et al. (2019) may also be interpreted as respective PID-5 scales having low discriminant validity.

Concerning the ability of the PID5BF+ to differentiate between patient groups with and without a borderline PD diagnosis, significant differences in Negative Affectivity and Disinhibition facet and domain trait scores were found, which is in line with the proposed trait associations for borderline PD in the *DSM-5* as well as with empirical findings on the association of PID-5 traits and borderline PD (Watters, Bagby, et al., 2019). The effect sizes of the comparison of mean scores between patient groups also reflected the severity of the mental health conditions, with borderline PD showing the highest difference in total mean score compared with the group with only one diagnosis from the internalizing spectrum. This is in line with Zimmermann et al. (2020) who demonstrated that PID5BF+ total scores can be used as an indicator of PD severity. Noteworthy was also the ability of the PID5BF+, particularly of the domains Negative Affectivity, Detachment and Psychoticism, to differentiate between mild and more severe mental health conditions of the internalizing spectrum without a PD diagnosis. This finding underlines the possible conceptualization of maladaptive personality traits as transdiagnostically informative variables in mental health.

### Limitations and Future Directions

A major limitation of our study concerns the lack of informant reports or interview data, which constitutes an important data source for validation, especially in the assessment of socially undesirable personality features. Furthermore, convergent and discriminant validity assessments are most likely biased toward 1 as the short and long versions of the scale have not been assessed separately, leading to inflated correlations. Moreover, we had more female than male participants in the three construction samples, although for the clinical sample, the female to male ratio was representative for this population. Further limitations concern the utility of the PID5BF+ as a standalone measure. Although our results show good reliability and validity, a 34-item measure cannot provide the diagnostic precision and coverage of a 220-item measure, especially with respect to the facet traits, that are assessed with only two items. Although we used several runs of ACO and compared the results by hand, ACO is an automatic method with the danger of overspecifity of the solution to the sample at hand. Therefore, further cross-cultural validation studies are needed to investigate its reliability as a standalone measure as well as its robustness in terms of temporal stability and occasion specificity. Further research is particularly needed on the domain of Anankastia. It had the lowest reliability among all six domains and the two underlying constructs of perseveration and rigid perfectionism showed remarkable differences especially in terms of correlations with Big Five Conscientiousness and PiCD Anankastia. One solution could be to remove perseveration and to integrate a broader set of items from rigid perfectionism (Bach, Kerber, et al., 2020). Another solution could be to expand the item scope beyond the PID-5 (Crego, Samuel, et al., 2015). A more general question concerns the construct validity of a separate Anankastia domain itself as recent exploratory factor analyses tend to find a 4 rather than 5-factor latent structure for the ICD-11 PD model with a bipolar dimension defined by Disinhibition and Anankastia (Bach, Christensen, et al., 2020; Carnovale et al., 2020).

Nevertheless, the results of this study suggest that the PID5BF+ can be utilized not only as a diagnostic measure for maladaptive personality traits according to *DSM-5* but also as an assessment basis for treatment planning (Hopwood, 2018a) and outcome monitoring. As an onboarding or intake measure, it provides important information for treatment planning and predictions about possible outcomes, while as an outcome assessment measure, it enables the tracking of changes in maladaptive traits which may be amenable through psychological interventions (Roberts et al., 2017). The hierarchical and dimensional assessment of psychopathology bears a huge opportunity for improvement in mental health care and research (Conway, 2019; Hopwood et al., 2019), and the routine application of the PID5BF+ might be a promising step in this direction.

## Supplemental Material

sj-pdf-1-asm-10.1177_1073191120971848 – Supplemental material for Development of a Short and ICD-11 Compatible Measure for DSM-5 Maladaptive Personality Traits Using Ant Colony Optimization Algorithms Click here for additional data file. Supplemental material, sj-pdf-1-asm-10.1177_1073191120971848 for Development of a Short and ICD-11 Compatible Measure for DSM-5 Maladaptive Personality Traits Using Ant Colony Optimization Algorithms by André Kerber, Martin Schultze, Steffen Müller, Rosa Maria Rühling, Aidan G. C. Wright, Carsten Spitzer, Robert F. Krueger, Christine Knaevelsrud and Johannes Zimmermann in Assessment
