# Supplementary material for: Development of a Short and ICD-11 Compatible Measure for DSM-5 Maladaptive Personality Traits Using Ant Colony Optimization Algorithms
Source: Assessment. 2020 Dec 28;29(3):467–87. doi: 10.1177/1073191120971848 (PMC8866743; doi:10.1177/1073191120971848)
Supplement: sj-pdf-1-asm-10.1177_1073191120971848 – Supplemental material for Development of a Short and ICD-11 Compatible Measure for DSM-5 Maladaptive Personality Traits Using Ant Colony Optimization Algorithms [file sj-pdf-1-asm-10.1177_1073191120971848.pdf]

Supplemental Materials

Development of a Short and ICD-11 compatible Measure for DSM-5 Maladaptive Personality Traits Using Ant Colony Optimization Algorithms

by A. Kerber, M. Schultze, S. Müller, R. M. Rühling, A. G. C. Wright, C. Spitzer, R. F. Krueger, C. Knaevelsrud and J. Zimmermann

Supplemental File 1

Setting of the Optimization Parameter in the two ACO runs:

First Algorithm

The individual optimization criteria were transformed based on logarithmic functions as follows:

$$f_{RMSEA}(s) = 1 - \frac{1}{1 + e^{-100(RMSEA_S - .05)}} \ ,$$

$$f_{SRMR}(s) = 1 - \frac{1}{1 + e^{-100(SRMR_S - .07)}} \ ,$$

$$f_{CFI}(s) = \frac{1}{1 + e^{-100\,(CFI_S - .95)}} \ ,$$

$$f_{rel}(s) = \frac{1}{1 + e^{-10(\overline{\omega_t} - .8)}}$$

The logistic function is chosen following the arguments presented by Schultze (2017): a sharper discrimination between solutions in areas of interest and imposing boundaries on the objective function, which are known a priori. Regarding the areas of interest for model fit criteria, the components of the objective function were defined such that they discriminate maximally around the common cut-off criteria used to separate adequately fitting models from those that are not tenable (i.e., RMSEA < .05, SRMR < .07, CFI > .95; Hu & Bentler, 1999; Marsh et al., 2005). These components of the objective function are combined to a composite pheromone value:

$$f_{composite}(s) = f_{rel}(s) + \frac{f_{RMSEA}(s) + f_{SRMR}(s) + f_{CFI}(s)}{3}$$

The algorithm converges if all composite item pheromones lie within a tolerance window of minimum and maximum values according to Stützle and Hoos (2000) or stops if a maximum

number of iterations is exceeded. We set the maximum number of iterations without improvements to 128, a maximum of 20 item selections were tested per iteration and the pheromone evaporation rate was set to .95.

Second Algorithm

As in the previous algorithm, we used a sum of logistic functions for the optimization criteria, which remained the same for RMSEA and CFI. We changed the turning point of the optimization function for the reliability from .8 to .7 and further added the following functions for the parameters for internal consistencies and the average correlation between short and long scale versions:

Concerning mean factor saturation in terms of McDonalds Omega:

$$f_{omega}(s) = \frac{1}{1 + e^{-10(\overline{\omega_t} - .7)}}$$

And minimum factor loadings:

$$f_{min_{lam}}(s) = \frac{1}{1 + e^{-10(min_{lams_s} - .4)}}$$

And average correlation between shortened and long versions across all facets and domains

$$f_{cor_{sl}}(s) = \frac{1}{1 + e^{-10(cor_{sl} \; - .90)}}$$

The composite pheromone values as calculated in the algorithm were:

$$f_{composite}(s) = \frac{f_{RMSEA}(s) + f_{CFI}(s)}{2} + \frac{f_{omega}(s) + f_{min_{lam}}(s)}{2} + f_{cor}\;(s)$$

In every iteration, 60 item combinations were assessed for the above-described optimization criteria, and item pheromone values were updated for items leading to better solutions utilizing a pheromone multiplicator of iteration number 0.2 in order not to disadvantage later iterations. The algorithm converged after 40 iterations without improvements in the pheromone values.

Supplemental Table S1

Model fit and Measurement invariance across three groups: German-speaking non-clinical sample (N=560), German-speaking clinical sample (N=683) and English-speaking non-clinical sample (N = 1653).

**Model**	χ^2 (*df*)	**CFI**	**RMSEA** [90% CI]	**SRMR**	**Model comp**	**ΔCFI**	**ΔRMSEA** (% CI Overlap)	**ΔSRMR**
M1: Configural Invariance	4332.1 (1485)	.944	.047 [.046-.049]	.059	--	--	--	--
M2: Equal item thresholds and intercepts	4401.3 (1502)	.943	.047 [.046-.048]	.059	M1	.001	.000 (100%)	.000
M3: Equal item thresholds, intercepts and first/second order factor loadings	4459.2 (1536)	.942	.047 [.046-.049]	.060	M1	.002	.000 (100%)	.001
M4: Equal item thresholds, intercepts, first/second order factor loadings and residual variances	4537.3 (1604)	.942	.046 [.045-.048]	.061	M1	.002	.001 (80%)	.002

Note. CFI = scaled comparative fit index, RMSEA = scaled root mean square error of approximation, SRMR = standardized root mean square residual. χ^2 , CFI and RMSEA were scaled according to Satorra & Bentler (2001).

Supplemental File 2
Norm values in the German validation sample

Men

Table 1: T-scaled norm values for the PID-5 negative affectivity domain

	<21 (n = 49)	21-30 (n = 72)	31-40 (n = 62)	41-50 (n = 72)	51-60 (n = 88)	>61 (n = 81)
0	30	33	37	31	33	33
0.17	36	38	40	36	38	40
0.33	41	42	41	44	43	45
0.5	43	45	43	46	45	49
0.67	47	46	48	49	49	52
0.83	47	46	48	49	49	52
1	50	52	50	54	54	55
1.17	50	52	50	54	54	55
1.33	53	57	58	59	57	59
1.5	57	58	61	62	58	62
1.67	57	58	61	62	58	62
1.83	59	61	64	65	61	68
2	64	63	67	67	63	68
2.17	67	72	68	69	66	68
2.33	67	72	68	69	66	68
2.5	70			72	73	72
2.67				72	73	72
2.83				72	73	72
3						

Note. T-values are based on the relative cumulative frequency distribution.

Table 2: T-scaled norm values for the PID-5 detachment domain

	<21 (n = 49)	21-30 (n = 72)	31-40 (n = 62)	41-50 (n = 72)	51-60 (n = 88)	>61 (n = 81)
0	37	35	39	38	37	36
0.17	41	39	41	40	39	41
0.33	46	43	45	43	43	46
0.5	48	45	48	49	48	50
0.67	52	48	51	50	51	54
0.83	52	48	51	50	51	54
1	53	53	57	53	56	57
1.17	53	53	57	53	56	57
1.33	54	58	61	58	62	62
1.5	58	60	62	60	64	64
1.67	58	60	62	60	64	64
1.83	65	65	63	64	66	68
2	67	66	67	64	67	
2.17	67	66	67	66	70	
2.33	67	66	67	66	70	
2.5		69	68	72	73	
2.67		72	71	72		
2.83		72	71	72		
3						

Note. T-values are based on the relative cumulative frequency distribution.

Table 3: T-scaled norm values for the PID-5 antagonism domain

	<21 (n = 49)	21-30 (n = 72)	31-40 (n = 62)	41-50 (n = 72)	51-60 (n = 88)	>61 (n = 81)
0	30	31	37	38	39	39
0.17	35	35	41	43	43	44
0.33	37	38	44	46	47	46
0.5	43	42	46	48	50	50
0.67	46	46	48	50	51	52
0.83	46	46	48	50	51	52
1	52	50	54	55	57	58
1.17	52	50	54	55	57	58
1.33	54	56	59	60	63	65
1.5	58	60	61	62	67	68
1.67	58	60	61	62	67	68
1.83	70	62	65	67	70	70
2	70	63	67	72		70
2.17		66	67	72		
2.33		66	67	72		
2.5		72	68			
2.67		72	71			
2.83		72	71			
3						

Note. T-values are based on the relative cumulative frequency distribution

Table 4: T-scaled norm values for the PID-5 disinhibition domain

	<21 (n = 49)	21-30 (n = 72)	31-40 (n = 62)	41-50 (n = 72)	51-60 (n = 88)	>61 (n = 81)
0	33	35	38	34	37	40
0.17	37	40	42	40	42	42
0.33	38	44	42	43	45	46
0.5	43	44	48	47	47	50
0.67	47	46	51	51	50	53
0.83	47	46	51	51	50	53
1	53	51	55	56	57	59
1.17	53	51	55	56	57	59
1.33	54	56	58	60	60	64
1.5	58	61	61	62	64	68
1.67	58	61	61	62	64	68
1.83	60	67	71	69	68	
2	63	67	71	69	73	
2.17	65	67	71	72		
2.33	65	67	71	72		
2.5		72	71	72		
2.67			71			
2.83			71			
3						

Note. T-values are based on the relative cumulative frequency distribution.

Table 5: T-scaled norm values for the PID-5 psychoticism domain

	<21 (n = 49)	21-30 (n = 72)	31-40 (n = 62)	41-50 (n = 72)	51-60 (n = 88)	>61 (n = 81)
0	30	31	38	35	37	38
0.17	35	33	40	39	42	41
0.33	41	40	45	43	45	46
0.5	44	44	48	46	47	49
0.67	50	47	49	48	50	52
0.83	50	47	49	48	50	52
1	52	52	53	55	55	56
1.17	52	52	53	55	55	56
1.33	55	58	58	60	60	61
1.5	56	60	62	62	65	64
1.67	56	60	62	62	65	64
1.83	62	66	64	66	73	67
2	64	67	67	67	73	72
2.17	67	67	71	69	73	72
2.33	67	67	71	69	73	72
2.5	70					
2.67	70					
2.83	70					
3						

Note. T-values are based on the relative cumulative frequency distribution.

Table 6: T-scaled norm values for the PID-5 anankastia domain

	<21<br>(n = 49)	21-30<br>(n = 72)	31-40<br>(n = 62)	41-50<br>(n = 72)	51-60<br>(n = 88)	>61<br>(n = 81)
0	33	31	32	36	34	38
0.25	33	40	35	40	41	41
0.5	39	43	42	42	45	45
0.75	44	47	46	47	47	49
1	48	51	49	50	53	53
1.25	51	54	54	55	56	57
1.5	55	59	59	60	60	62
1.75	58	62	63	62	67	65
2	64	66	68	67	70	68
2.25	67	69	71	72	73	
2.5		72	71	72		
2.75						
3						

Note. T-values are based on the relative cumulative frequency distribution.

Women

Table 1: T-scaled norm values for the PID-5 negative affectivity domain

	<21 (n = 49)	21-30 (n = 71)	31-40 (n = 75)	41-50 (n = 75)	51-60 (n = 90)	>61 (n = 58)
0			35	36	32	40
0.17	35	33	37	39	36	42
0.33	35	38	38	41	39	46
0.5	36	40	41	43	43	49
0.67	37	44	43	46	46	54
0.83	37	44	43	46	46	54
1	42	50	48	51	51	56
1.17	42	50	48	51	51	56
1.33	46	53	54	56	55	58
1.5	49	54	56	58	58	62
1.67	49	54	56	58	58	62
1.83	51	57	58	65	63	68
2	56	60	62	68	66	71
2.17	56	62	64	72	68	71
2.33	56	62	64	72	68	71
2.5	61	66	72		73	71
2.67	64	67	72		73	
2.83	64	67	72		73	
3						

Note. T-values are based on the relative cumulative frequency distribution.

Table 2: T-scaled norm values for the PID-5 detachment domain

	<21 (n = 49)	21-30 (n = 71)	31-40 (n = 75)	41-50 (n = 75)	51-60 (n = 90)	>61 (n = 58)
0	33	35	38	38	37	43
0.17	38	41	43	42	43	45
0.33	42	45	47	46	48	47
0.5	44	48	50	49	50	50
0.67	48	50	51	51	53	51
0.83	48	50	51	51	53	51
1	52	53	57	56	57	58
1.17	52	53	57	56	57	58
1.33	54	58	61	58	63	63
1.5	56	59	62	59	66	65
1.67	56	59	62	59	66	65
1.83	62	61	69	65	68	68
2	64	67	69	66	70	71
2.17	65	72	72	69	73	71
2.33	65	72	72	69	73	71
2.5	70	72		72		
2.67	70					
2.83	70					
3						

Note. T-values are based on the relative cumulative frequency distribution.

Table 3: T-scaled norm values for the PID-5 antagonism domain

	<21 (n = 49)	21-30 (n = 71)	31-40 (n = 75)	41-50 (n = 75)	51-60 (n = 90)	>61 (n = 58)
0	33	39	37	37	40	41
0.17	38	41	40	41	45	48
0.33	42	44	45	46	49	53
0.5	44	45	49	49	54	56
0.67	47	48	51	53	57	58
0.83	47	48	51	53	57	58
1	53	53	57	57	63	71
1.17	53	53	57	57	63	71
1.33	58	58	64	64	67	
1.5	60	61	69	69	70	
1.67	60	61	69	69	70	
1.83	64	66		72		
2	65	69				
2.17	67					
2.33	67					
2.5						
2.67						
2.83						
3						

Note. T-values are based on the relative cumulative frequency distribution..

Table 4: T-scaled norm values for the PID-5 disinhibition domain

	<21	21-30	31-40	41-50	51-60	>61
	(n = 49)	(n = 71)	(n = 75)	(n = 75)	(n = 90)	(n = 58)
0	30	33	38	38	39	40
0.17	36	40	39	42	43	45
0.33	41	44	43	44	47	49
0.5	42	47	44	47	49	50
0.67	47	50	50	51	53	54
0.83	47	50	50	51	53	54
1	51	56	57	55	58	60
1.17	51	56	57	55	58	60
1.33	55	58	61	61	64	65
1.5	58	60	62	63	67	66
1.67	58	60	62	63	67	66
1.83	67	64	72	66	67	71
2	67	67		68	73	
2.17	70	72		69		
2.33	70	72		69		
2.5	70	72				
2.67	70	72				
2.83	70	72				
3						

Note. T-values are based on the relative cumulative frequency distribution.

Table 5: T-scaled norm values for the PID-5 psychoticism domain

	<21 (n = 49)	21-30 (n = 71)	31-40 (n = 75)	41-50 (n = 75)	51-60 (n = 90)	>61 (n = 58)
0	30	37	34	38	35	43
0.17	37	41	42	43	42	46
0.33	41	43	46	45	47	48
0.5	44	47	49	46	51	51
0.67	46	51	50	49	53	54
0.83	46	51	50	49	53	54
1	49	55	54	57	55	61
1.17	49	55	54	57	55	61
1.33	54	60	61	62	59	64
1.5	55	62	61	66	62	71
1.67	55	62	61	66	62	71
1.83	58	67	65	72	67	
2	61		68		73	
2.17	64		69			
2.33	64		69			
2.5	65		72			
2.67	70					
2.83	70					
3						

Note. T-values are based on the relative cumulative frequency distribution.

Table 6: T-scaled norm values for the PID-5 anankastia domain

	<21 (n = 49)	21-30 (n = 71)	31-40 (n = 75)	41-50 (n = 75)	51-60 (n = 90)	>61 (n = 58)
0	30	34	38	34	36	41
0.25	37	37	41	40	40	45
0.5	39	44	43	43	44	50
0.75	42	47	47	47	47	52
1	48	51	52	51	54	55
1.25	50	55	54	57	58	58
1.5	52	60	59	62	62	63
1.75	58	63	64	66	66	68
2	64	67	68		70	
2.25	67	72	72		73	
2.5	70	72	72			
2.75	70	72				
3						

Note. T-values are based on the relative cumulative frequency distribution.
